# Spinal Cord Ventral Horns and Lymphoid Organ Involvement in Powassan Virus Infection in a Mouse Model

**DOI:** 10.3390/v8080220

**Published:** 2016-08-12

**Authors:** Rodrigo I. Santos, Meghan E. Hermance, Benjamin B. Gelman, Saravanan Thangamani

**Affiliations:** 1 Department of Pathology, University of Texas Medical Branch, Galveston, TX 77555, USA; meherman@UTMB.EDU (M.E.H.); bgelman@UTMB.EDU (B.B.G.); sathanga@UTMB.EDU (S.T.); 2 Institute for Human Infections and Immunity, University of Texas Medical Branch, Galveston, TX 77555, USA; 3 Galveston National Laboratory, University of Texas Medical Branch, Galveston, TX 77555, USA

**Keywords:** Powassan virus, Poliomyelitis-like syndrome, lymphoid organs infection

## Abstract

Powassan virus (POWV) belongs to the family *Flaviviridae* and is a member of the tick-borne encephalitis serogroup. Transmission of POWV from infected ticks to humans has been documented in the USA, Canada, and Russia, causing fatal encephalitis in 10% of human cases and significant neurological sequelae in survivors. We used C57BL/6 mice to investigate POWV infection and pathogenesis. After footpad inoculation, infected animals exhibited rapid disease progression and 100% mortality. Immunohistochemistry and immunofluorescence revealed a very strong neuronal tropism of POWV infection. The central nervous system infection appeared as a meningoencephalitis with perivascular mononuclear infiltration and microglial activation in the brain, and a poliomyelitis-like syndrome with high level of POWV antigen at the ventral horn of the spinal cord. Pathological studies also revealed substantial infection of splenic macrophages by POWV, which suggests that the spleen plays a more important role in pathogenesis than previously realized. This report provides a detailed description of the neuroanatomical distribution of the lesions produced by POWV infection in C57BL/6 mice.

## 1. Introduction

Powassan virus (POWV) is a tick-borne flavivirus [1,2] transmitted by *Ixodes scapularis* ticks to small or medium sized mammals and accidentally to humans as a spillover from the main infection cycle [1]. Antibody neutralization analysis, in situ hybridization, and genotyping place POWV within the tick-borne encephalitis virus (TBEV) serogroup [3,4,5,6]. POWV is pathogenic for humans, and 10% of reported cases are fatal [1] with mortality reaching 36% in a series of cases observed in New York, USA [7]. The scarce number of epidemiological studies emphasizes the underestimation of POWV mortality ratio. In patients who develop encephalitis the fatality rate is about 60%. Neurological sequelae are observed in half of the survivors [8,9,10]. POWV was first isolated from the brain of a 5-year-old boy who died in 1958 of encephalitis [11]. Since then the number of human cases has steadily increased, [12] suggesting that POWV is an emerging disease or the recognition of cases have increased. According to the Center for Disease Control and Prevention (CDC), approximately 60 cases of POWV have been documented in the past 10 years in the USA [13].

POWV encephalitis symptoms start after an incubation period that typically ranges from eight to 34 days after inoculation. Initial signs are non-specific and can include fever, sore throat, sleepiness, disorientation, and headaches. POWV encephalitis is characterized by vomiting, respiratory distress, convulsions and long-lasting fever. The encephalopathy described for POWV includes general weakness, ataxia, tremors and respiratory failure in the more severe cases. Lethargy and paralysis are usually observed and hemiplegia is the most common form of paralysis [1,7,8,9,14]. Brain autopsy results have revealed dense perivascular and parenchymal inflammatory infiltration. Neurons in the brainstem, cerebellar Purkinje cells, basal ganglia, and thalamus were infected in most human cases that underwent an autopsy. Viral antigens and/or viral RNA were demonstrated within central nervous system (CNS) neurons, suggesting a strong neurotropy [8,9,10,11]. Spinal cord necropsy reports are scarce for POWV infections. McLean and Donohue [11] demonstrated monocyte and lymphocyte infiltrates in the spinal cord. A more detailed analysis of spinal cord infection by deer-tick virus encephalitis (DTV, POWV lineage II), showed mononuclear infiltrates accentuated in the anterior horns; the presence of DTV was confirmed by sequencing [8].

Infection of animals with POWV is also characterized by neuronal tropism. Lesions on non-human primates infected intracerebrally with POWV are mainly inflammatory and degenerative, marked in the cortex, cerebellum and spinal cord, and have a strong presence of virus in neurons [15]. Mice infected with the Russian P-40 strain of POWV revealed a destructive inflammatory disease in all parts of the brain. Viral particles were detected by electron microscopy in the perikarion of neurons and in glial cells [16]. Histopathologically, infected mice display neuron loss, perivascular lymphocytic cuffing, and mononuclear cell infiltration akin to what has been observed in human infection. Clinical signs in POWV-infected mice included hyperresponsiveness, ruffled fur, malaise, hunched posture, ataxia, loss of balance and paralysis [17,18]. Other viruses from the tick-borne encephalitis complex are also highly neurotropic [19]. After peripheral inoculum of TBEV (Oshima strain) virus loads at the brain reached titers above 10^6^ PFU/g on the 5th day pi and immunohistochemistry staining indicated infected neurons [20]. Studies on mouse [21] and human [22] neuron primary cultures suggested that the TBEV infection is responsible for neuron morphological changes and viral accumulation in neuronal extensions/dendrites. 

This study used histological techniques to elucidate POWV pathogenesis in the CNS, and also in lymphoid and nonlymphoid organs including the liver, kidney, pancreas, and muscle. The footpad injection performed in this study is a route of entry that mimics the transcutaneous tick feeding process. We found perivascular infiltration of mononuclear cells and an intense infection of neurons in the brain, as demonstrated in previously described models. We also demonstrated a poliomyelitis-like syndrome caused by the infection of anterior horn cells in the spinal cord. Additionally, we demonstrated that infection of the spleen and lymph nodes are important in the pathophysiology of POWV.

## 2. Materials and Methods 

### 2.1. Animals and POWV Infection

Four-week-old male C57BL/6 mice were purchased from Jackson Laboratories (Ben Harbor, ME, USA). All mouse experiments were conducted in accordance with an animal use protocol approved by the University of Texas Medical Branch (UTMB) Institutional Animal Care and Use Committee (IACUC). A group of eight mice were inoculated with 5 × 10^6^ PFU, another group of eight mice were inoculated with 5 × 10^3^ PFU and one additional group with four animals was mock infected with Minimum Essential Medium (MEM) supplemented with 2% fetal bovine serum (FBS) and was used as a control in all experiments. All inoculums were given in the hind footpad and had approximately 70 μL of volume. All animals were weighed and blood samples were collected daily. Clinical symptoms and behavioral alterations were monitored daily. All infections were conducted with POWV LB strain (stock passed seven times in Vero cells). 

Statistical significance for weight progression was accessed using factorial analysis of variance (ANOVA – FACTORIAL, Software Statistica version 7.0). For the mortality curve, statistical analysis was performed using the chi-square test. For both, statistical significance was accepted with *p* < 0.05.

### 2.2. Ethics

Research was conducted in compliance with the Animal Welfare Act and other federal statutes and regulations relating to animals and experiments involving animals, and adhered to principles stated in the eighth edition of the Guide for the Care and Use of Laboratory Animals, National Research Council, 2013 [23]. Research described in this manuscript was approved by the UTMB Institutional Animal Care and Use Committee (IACUC, reference # 0907054B). All steps were taken to ameliorate pain and distress in accordance with the Guide for the Care and Use of Laboratory Animals, National Research Council, 2013 and the approved UTMB IACUC animal use protocol. Animals were housed under controlled conditions of humidity, temperature, and light (12-h light/12-h dark cycles). Food and water were available ad libitum. Animals were monitored no less than twice daily by trained personnel. All procedures were conducted by properly trained personnel under the oversight of an attending veterinarian and the principal investigator. Humane endpoint criteria was specified and approved by the UTMB IACUC and strictly adhered to. Specifically, we applied a scoring sheet assessing ruffled fur, respiratory distress, weakness, changes in behavior and/or paralysis. The animals on this protocol were euthanized by CO_2_ inhalation when they met criteria for euthanasia or at the study endpoint as approved.

### 2.3. Virus Titration

The blood was stored at −80 °C until a PFU assay was performed. For the PFU assay, 200 μL of total blood 10-fold serial dilutions was infected in monolayers of BHK-SA cells grown in 12-well plates in the presence of MEM supplemented with 2% FBS and Tragacanth (Sigma-Aldrich, St. Louis, MO, USA) for overlay. The plates were incubated at 37 °C in the presence of 5% CO_2_. On the third day, the presence of plaques was evaluated using crystal violet staining (diluted in formalin) and expressed as PFU/mL [24]. Student’s *t*-test was used to check for statistical significance. Statistical significance was accepted at *p* < 0.05.

### 2.4. Histopathology and Immunohistochemistry (IHC)

Brain, spinal cord (whole vertebra), liver, spleen, pancreas, and kidney were extracted and formalin-fixed for 48 h. Vertebrae were treated for 6 h in decalcifying solution from Thermo Scientific (Waltham, MA, USA) for decalcification. The tissues were dehydrated in increasing ethanol concentration, treated in xylenes, and paraffin-embedded [25]. Five micron paraffin sections were deparaffinized in xylenes and re-hydrated in decreasing concentrations of ethanol. A few selected slides were stained with hematoxylin–eosin (HE) following routine protocols [25,26], and examined under light microscopy. For the detection of viral antigens, deparaffinized slides were rehydrated in ethanol, and subjected to DAKO Target Retrieval Solution (Dako North America Inc., Carpinteria, CA, USA) for 15 min with microwave heating [27]. After the slides reached room temperature they were incubated 30 min in 4% H_2_O_2_ for endogenous peroxidase quenching. Mouse-On-Mouse kit (MOM—Vector labs, Burlingame, CA, USA) was used to overcome the high background generated when mouse antibodies are used in mouse tissue [26,28]. Briefly, slides were incubated with MOM mouse IgG blocking solution for 1 h. Hyper Mouse Immune Ascitic Fluid (HMIAF) antibody against POWV (kindly provided by Dr. Robert Tesh from UTMB, TX, USA) was diluted 1:300 (in MOM diluent) and incubated for 30 min at room temperature. MOM biotinylated horse anti-mouse antibody was used as secondary antibody. The detection of biotinylated antibody was performed with Streptavidin-peroxidase Ultrasensitive Polymer (Sigma-Aldrich) [29] followed by NovaRed (Vector labs, Burlingame, CA, USA) chromogen staining. Slides were counterstained with Harris hematoxylin and mounted in Permount (Fisher Scientific, Hamptom, NH, USA) [30].

For POWV detection in the spleen the same protocols were used with the following exceptions: biotin blocking (Dako North America Inc.) was performed prior to the MOM blocking, the anti-POWV HMIAF was diluted 1:600 instead of 1:300, and the secondary antibody was diluted 1:600 instead of 1:400. 

### 2.5. Immunohistochemistry for GFAP and CD11b

For Glial Fibrillary Acidic Protein (GFAP) and CD11b staining, slides were subjected to alkaline antigen retrieval using 10 mM Tris solution with 0.05% Tween 20 (pH 10) and microwave heating for 15 min [27]. After slides reached room temperature, peroxidase blocking using 4% H_2_O_2_ was performed. Sections were blocked for 60 min in 5% goat serum. The primary antibody (GFAP rabbit antibody (Millipore, Temecula, CA, USA); CD11b, rabbit antibody (Abcam, Cambridge, MA, USA)) was diluted 1:100 for both CD11b and GFAP reactions and incubated for 1 h at 37 °C. Slides were washed and incubated with biotinylated goat anti-rabbit immunoglobulin (1:500) (Dako North America Inc.) for 30 min at room temperature [30]. The biotin reaction, staining, and counterstaining were executed as described above. All serum and antibodies were diluted in phosphate-buffered saline (PBS)-bovine serum albumin (BSA)-0.1% -Triton X100, pH 7.4.

### 2.6. Immunofluorescence (IF) in Paraffin Sections

Hydration and endogenous peroxidase quenching of tissue sections were done as described above. Samples were irradiated with UV light for 60 min for autofluorescence photobleaching [31] and treated an additional 30 min in 0.5 M glycine for free aldehyde binding. Tissue IgG was blocked using MOM blocking solution [26,28] with 5% goat serum. HMIAF antibody against POWV was diluted 1:300 in the presence of chicken IgY antibodies against neuron specific nuclear protein (NeuN, 1:200 (Millipore, Temecula, CA, USA)) [32] and rabbit IgG against GFAP (1:100) [33] and incubated for 30 min at room temperature. MOM biotinylated horse anti-mouse (1:400), goat anti-IgY AlexaFluor 546 conjugated (1:200), and goat anti-rabbit IgG Alexafluor 488 conjugated (1:200) were used as secondary antibodies. Streptavidin conjugated with Alexafluor 647 (1:200) was used to detect the biotin present in the anti-mouse antibody. The counterstaining was performed with DAPI. The images were acquired in an Olympus BX61 upright epifluorescence microscope. All Alexafluor conjugates are from Life Technologies (Thermo Scientific, Waltham, MA, USA).

### 2.7. Dual Staining Protocols

Hydration and endogenous peroxidase quenching of tissue sections were done as described above. Multiple stains were performed in the very same section. The first staining consisted of a MOM reaction for POWV detection. POWV detection in spleen tissue was executed as described above except that the ethanol-soluble substrate AEC was used and mounting in water based mounting media (Vectamount, Vector labs, Burlingame, CA, USA) was done. To proceed to the second reaction the coverslip was removed by submerging slides in DI water and slides were dehydrated in increasing concentrations of ethanol to remove the AEC substrate [34]. Prior to the immunoreaction, the slides were subjected to peroxidase quenching [35,36] and biotin blocking to avoid remnants of staining from the first reaction. For the second reaction, alkaline antigen retrieval was performed (10 mM Tris, 0.05% Tween 20, pH 10). After cooling, 5% goat serum was used as antigen block (60 min incubation) followed by anti-F4/80 rabbit antibody (Thermo Scientific, Waltham, MA, USA) diluted 1:100 and incubated for 60 min at 37 °C. Goat anti-rabbit biotinylated antibody was used for detection. Avidin/HRP and AEC staining were performed as for the first reaction. After endogenous peroxidase quenching, biotin blocking, and antigen blocking, the third reaction was started. No retrieval was used in this step. Anti-CD11c hamster antibody (Thermo Scientific, Waltham, MA, USA) was used as primary antibody and anti-hamster biotinylated antibody was used for primary antibody detection. The remaining steps were the same as for reactions 1 and 2 [30]. For reaction 1 serum and antibodies were diluted in MOM diluent; for reactions 2 and 3 serum and antibodies were diluted in PBS–BSA-0.1% -Triton X100, pH 7.4. Anti-rabbit and anti-hamster biotinylated antibodies were Life Technologies products. 

### 2.8. TUNEL Assay (Terminal Deoxynucleotidyltransferase-Mediated dUTP Nick End-Labeling)

Slides were hydrated and prepared as described above. Tissue sections were washed four times with PBS and then subjected to TUNEL assay for apoptosis, using ApopTag Peroxidase in situ apoptosis detection kit (Millipore, Temecula, CA, USA). Tissue sections were incubated with the deoxynucleotidyltransferase (TDT) mix for 1 h at 37 °C. Digoxigenin-labeled nucleotides were added to the fragmented DNA. Peroxidase conjugated anti-digoxigenin antibody was used to detect labeled nucleotides [37,38] and DAB was used as reaction chromogen. After counterstaining with Harris hematoxylin, samples were dehydrated, treated in xylenes and mounted in Permount (Fisher Scientific, Hamptom, NH, USA).

## 3. Results

### 3.1. Clinical Observations

**Figure 1:**
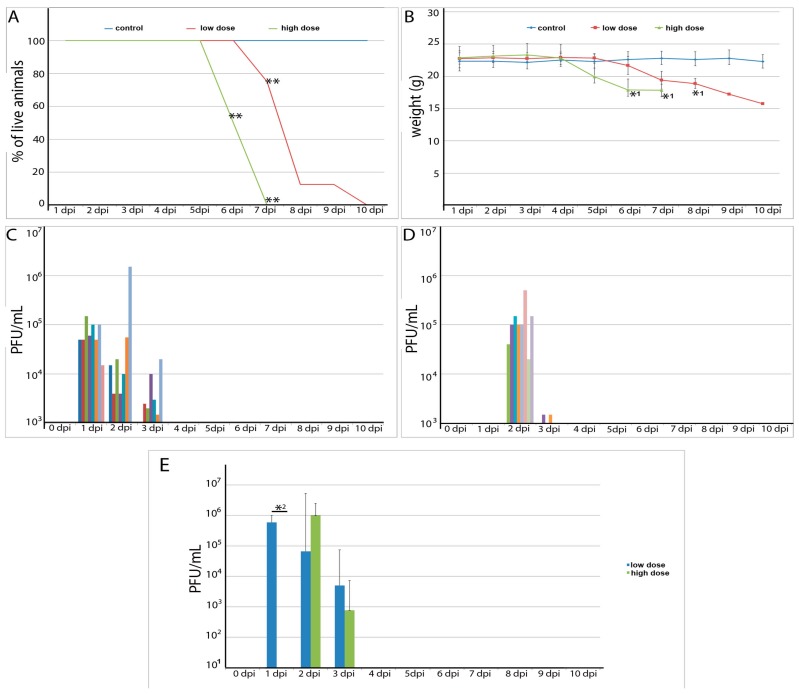
Clinical findings and viremia in murine Powassan virus (POWV) infection. (**A**) Mortality: control and POWV infected groups are shown (** significance with *p* < 0.0001); (**B**) Weight loss: control and POWV-infected groups are shown (significance with *p* < 0.05); (**C**) Viral load in the blood for POWV-infected group (infectious dose 5 × 10^3^ PFU; low dose). Each color represents one individual animal; (**D**) Viral load in the blood for POWV-infected group (infectious dose 5 × 10^6^ PFU; high dose). Each color represents one individual animal (**E**) Viral load average for high and low dose infection groups; * 2—significance with *p* < 0.0002.

Infection of mice with POWV resulted in 100% mortality with a rapid disease progression. Mock-infected animals remained active throughout the experiment. Animals inoculated with 5 × 10^6^ PFU (high dose group) presented severe sickness at 6 days post infection (dpi) and by 7 dpi all animals became moribund (Figure 1A). The mortality curve indicated significance at the 6 dpi and 7 dpi comparing the control group with the POWV infected group and at 8 dpi all animals succumbed to the disease (Figure 1A). In animals inoculated with 5 × 10^3^ PFU (low dose group), 3 mice were moribund at 7 dpi, 4 mice were moribund at 8 dpi, and 1 mouse survived until 10 dpi. The mortality curve indicated significance at the 6 dpi comparing the control and the low dose group. At 8 dpi all high dose animals were dead, however the significant difference between low dose and control remained (Figure 1A).

Mice inoculated with the POWV began to lose weight between days 5 and 7 post infection (Figure 1B). The onset of clinical manifestations was abrupt. The first signs included ruffled fur and behavioral alterations such as irritability or nonresponsiveness. The signs quickly evolved to hunched posture, and paralysis. Obvious neurological signs were observed. Flaccid paralysis was the most remarkable neurological alteration observed and was present in 7 out of 8 POWV infected mice in the high dose group. Other neurological signs of POWV infection included weak grip, altered reflex when grabbed by the tail, or walking/positioning abnormalities. Half of the animals in the low dose of infection group did not present paralysis; their symptoms included shaking, irresponsiveness and weight loss. The other half presented the same neurological signs observed for the animals inoculated with 5 × 10^6^ PFU. The neurological signs allied to the paralysis found in most animals, suggesting encephalomyelitis as the most reasonable hypothesis to be investigated.

### 3.2. Viremia

Viremia was observed in both the high and low dose groups. In the low dose group viremia was detected as early as 1 dpi, and in the high dose group viremia was detected as early as 2 dpi. Moreover, the low dose of infection group also presented a longer lasting viremia than the high dose infection group. (Figure 1C–E).

### 3.3. Histopathological Observations

#### 3.3.1. Central Nervous System

**Figure 2:**
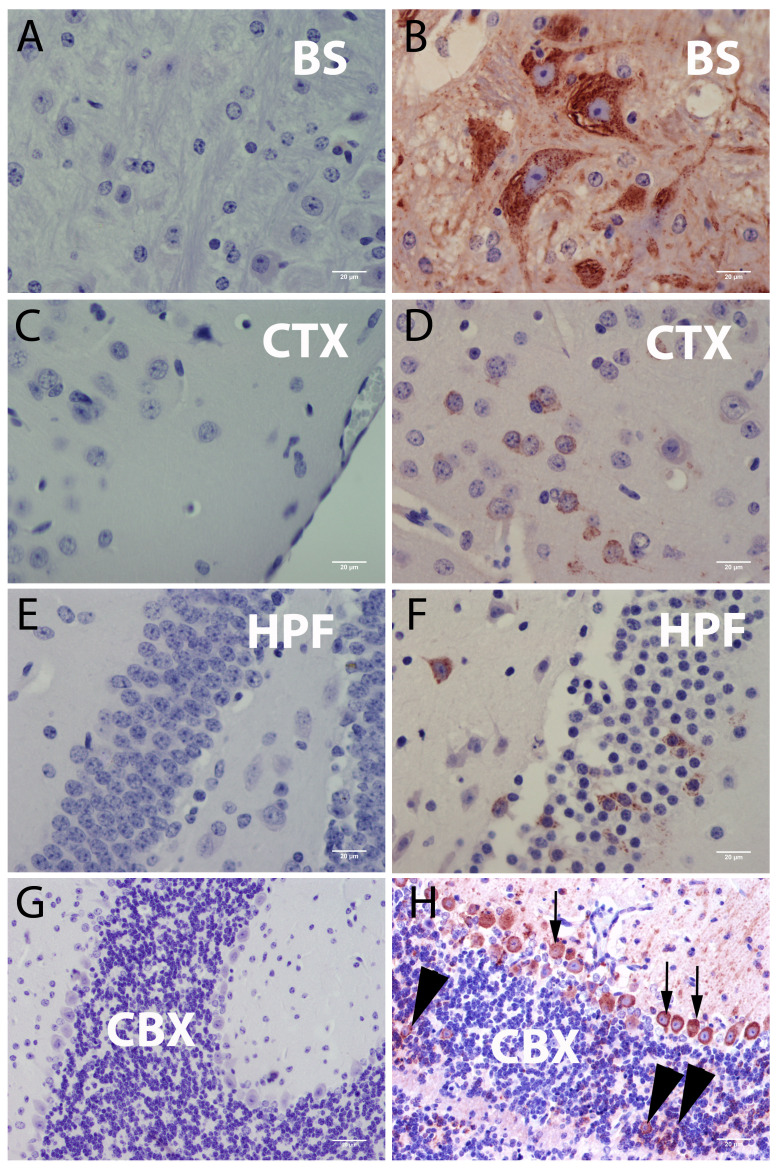
Immunohistochemistry for POWV antigen in mouse brain sections. (**A**) Brainstem (BS)—control animal; (**B**) Positively stained cells in the brainstem; (**C**) Neocortex (CTX)—control animal (**D**) Positively stained cells in the neocortex (**E**) Hippocampus (HPF)—control animal; (**F**) Infected cells and neuron vacuolization in the hippocampus; (**G**) Cerebellum (CBX)—control animal; (**H**) Cerebellum—POWV infected animal; (**H**) positively stained cells in the cerebellum (CBX), black arrowheads point to positive granular neurons and black arrows point to positive Purkinje cells. Scale bar corresponds to 20 μm (400× magnification). All animals presented in this figure were inoculated with 5 × 10^6^ PFU and developed clinical signs between the days 5 and 6 post infection.

The CNS was the main target in POWV infected mice. All animals had viral antigen detectable in both the brain (Figure 2A–H and Table S1) and spinal cord. POWV staining was most evident in brainstem neurons (15 out 16 infected animals) (Figure 2A,B) and in the thalamus (13 out 16 infected animals, Table S1). The cerebellum was infected in seven mice infected with 5 × 10^6^ PFU and in 3 animals infected with 5 × 10^3^ PFU. Cerebellar Purkinje neurons were consistently infected whereas the granular neurons were sparsely infected in a few animals (Figure 2G,H; Table S1).

**Figure 3:**
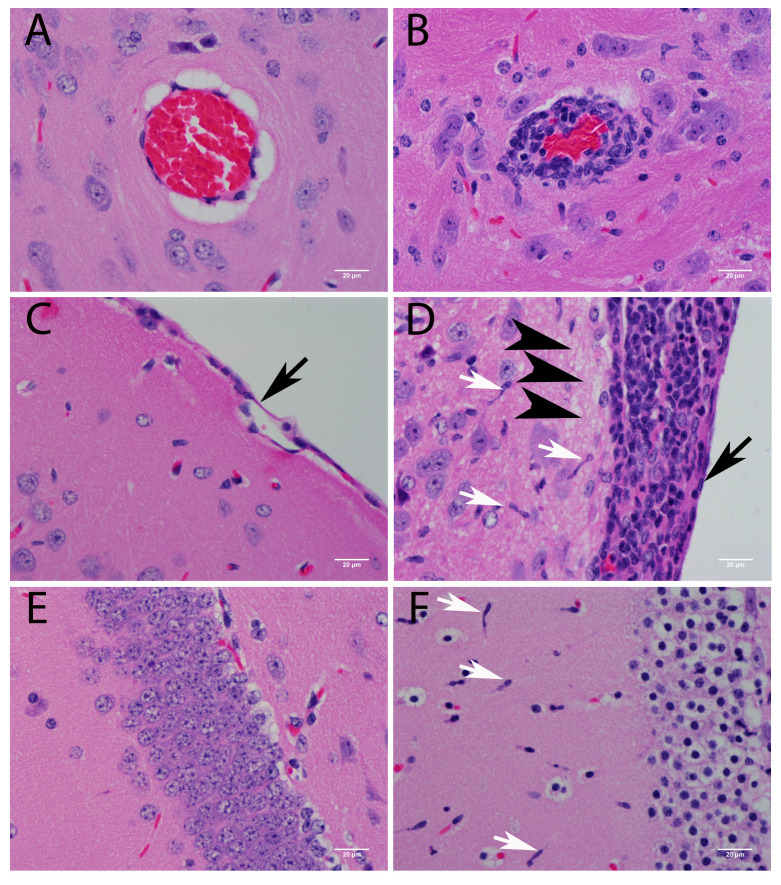
Histopathological findings in the brain of POWV-infected mice (hematoxylin–eosin (HE) staining). (**A**) Control animal: absence of inflammation around the vessel; (**B**) POWV-infected animal: perivascular infiltration; (**C**) Control animal, normal meninges; (**D**) POWV-infected animal: basal meningitis and vacuolization of the neuropil close to the leptomeninges (**E**) Control animal: normal hippocampus; (**F**) POWV-infected animal: hippocampus; Scale bars correspond to 20 μm (400× magnification). White arrows point to microglial cells (rod-cells), black arrows point to the meninges of control animals (**C**) and infected animals (**D**); Arrowheads point to vacuolization of the neuropil (**F**).

A prominent feature observed in this model was that perivascular mononuclear cell inflammatory infiltration was present throughout the brain (Figure 3A,B). Microglial cell activation was observed as a high density of rod-shaped cell nuclei in HE stained tissue sections. Rod-cells were especially numerous in neocortical lamina I close to the glia limitations and leptomeninges (Figure 3C–F). Leptomeningitis was present in all animals (Figure 3C,D) and was most consistently observed in the basal sectors of the cerebrum. Choroid plexitis, periventriculitis, and ventriculitis were present in a few animals. Commissural white matter fibers in the corpus callosum seldom if ever contained any POWV-stained cells (data not shown). Regardless of the dose used, all animals presented similar histopathological features at the CNS.

GFAP is a well-known marker used for astrocyte detection in the CNS [33] and is highly expressed when astrocytes are activated. CD11b is a general marker present in all myeloid cell lines [39]. The importance of macrophages, microglial cells and mononuclear cells during POWV infection in the brain was highlighted using CD11b immunohistochemistry. Stained cells were observed throughout the brains of these animals. The CD11b stain highlighted the increased number of microglia cells and the high number of recruited macrophages (Figure S1). Microglia were immunostained for CD11b less strongly than recruited macrophages (Figure S1). Astrocytes become hypertrophied in response to virtually all CNS disturbances and are referred to generally as astrogliosis. Hypertrophied astrocytes contain abundant GFAP, which makes GFAP staining a suitable histological marker for astrogliosis. As expected, CNS specimens from all of the infected animals contained astrogliosis (Figure S2). Heightened GFAP expression was not restricted to infected areas and was widespread throughout the brain. GFAP staining in the brainstem and hippocampus was notably strong and was present in all infected animals (Figure S2B,D). 

**Figure 4:**
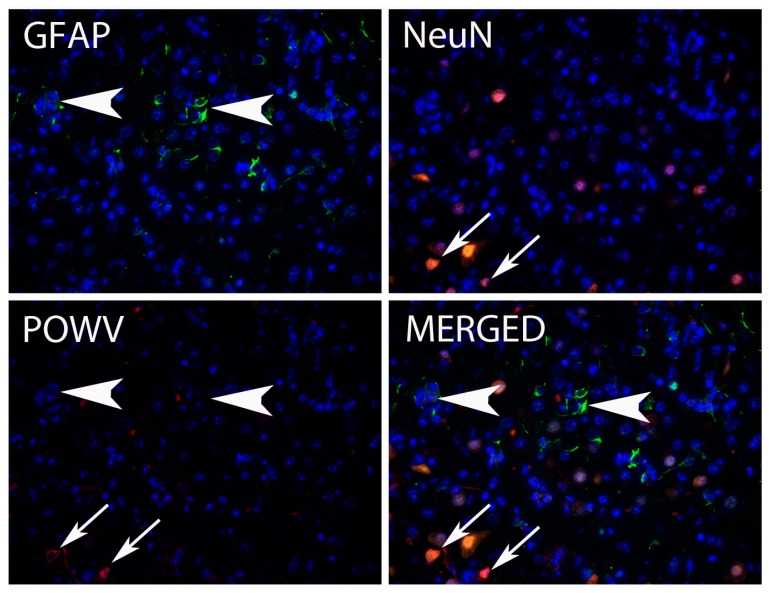
Immunofluorescence for Glial Fibrillary Acidic Protein (GFAP), NeuN, and POWV detection in brain sections. Astrocyte marker GFAP is shown in green; Neuron marker NeuN is shown in orange and POWV-positive cells are shown in red. Arrows point to POWV-positive neurons and arrowheads point to GFAP-positive cells. Note that GFAP positive-cells do not present POWV-staining. (400× magnification).

Histologically, neurons were greatly affected by POWV infection. In HE stained sections, neuropil vacuolization was a common finding associated with viral infection (Figure 3D). Pathological analysis has suggested that neurons are the main target for CNS POWV infection, and results of immunofluorescence microscopy (IFA) solidly confirmed that in this study. IFA showed POWV stain present in NeuN positive cells (an antigen specific for neuronal nuclei), but not cells stained for the astrocyte marker GFAP (Figure 4). POWV antigen was present in neuronal perikarya and not in neuronal nuclei.

**Figure 5:**
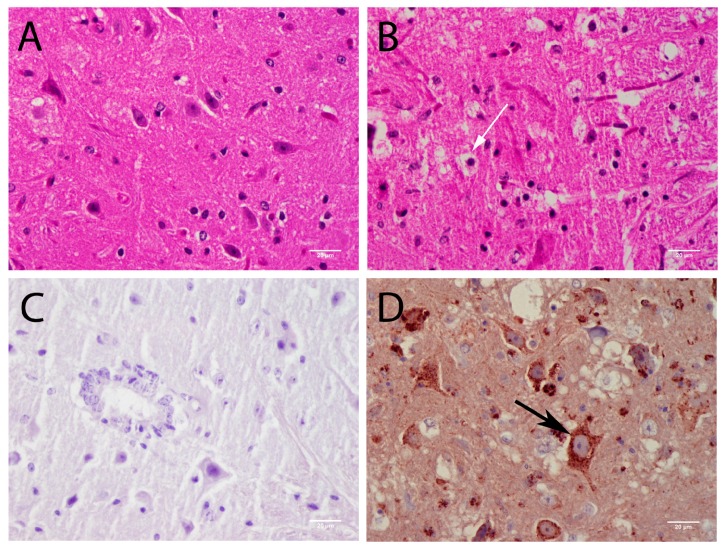
POWV histopathology in the spinal cord. (**A**) control animal: HE staining (400× magnification); (**B**) POWV-infected animal: HE staining (400× magnification); (**C**) Control animal: POWV immunostaining (400× magnification); (**D**) Widespread POWV antigen immunostaining (400× magnification); scale bars correspond to 20 μm (400× magnification, black arrow indicates infected motor neuron. White arrow indicates vacuolization of the neuropil.

In the spinal cord, large motor neurons in the ventral horns were mainly affected (Figure 5A–D). Consistent with the other CNS structures described above, meningitis and rod cells also were observed in the spinal cord. The intensity of spinal cord infection was highly variable between animals. Some animals had widespread infection of the spinal cord (Figure 5D) with widespread vacuolization of the neuropil; other animals had infection restricted to the ventral area of the spinal cord. Dorsal root ganglia (DRG) neurons did not stain for POWV antigen in all of the infected animals, which is consistent with predominantly motor neuron involvement at the level of the spinal cord.

#### 3.3.2. Lymphoid Organs

**Figure 6:**
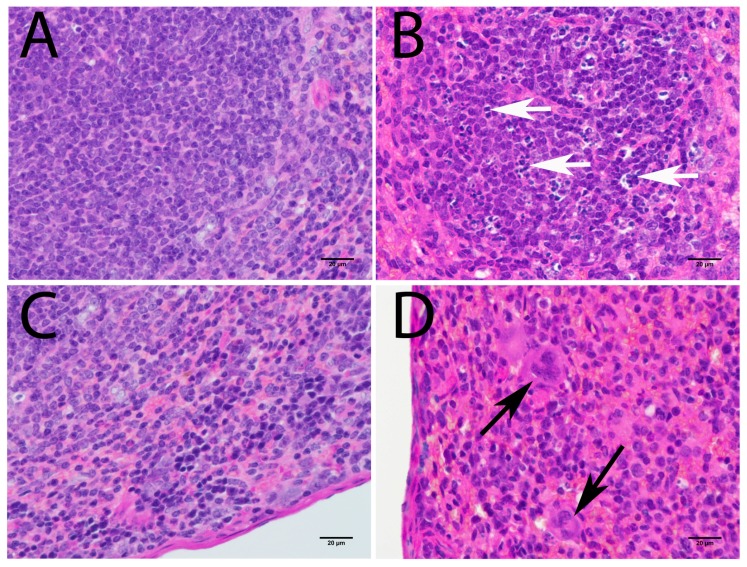
Histopathological findings in the spleen (H & E staining). (**A**) Control animal: white pulp; (**B**) POWV-infected animal: white pulp (**C**) Control animal: subcapsular area; (**D**) POWV-infected animal: subcapsular area. White arrows point to tingible body macrophages; black arrows point to giant cells. Scale bar corresponds to 20 μm (400× magnification).

**Figure 7:**
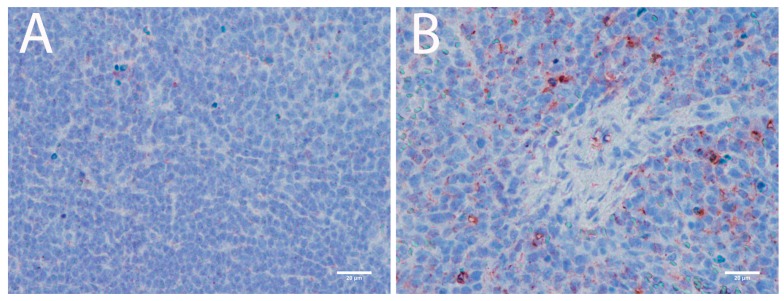
Immunohistochemistry for POWV shows that the virus has infected spleen. (**A**) Control animal; (**B**) POWV-infected animal. The scale bar corresponds to 20 μm (400× magnification).

**Figure 8:**
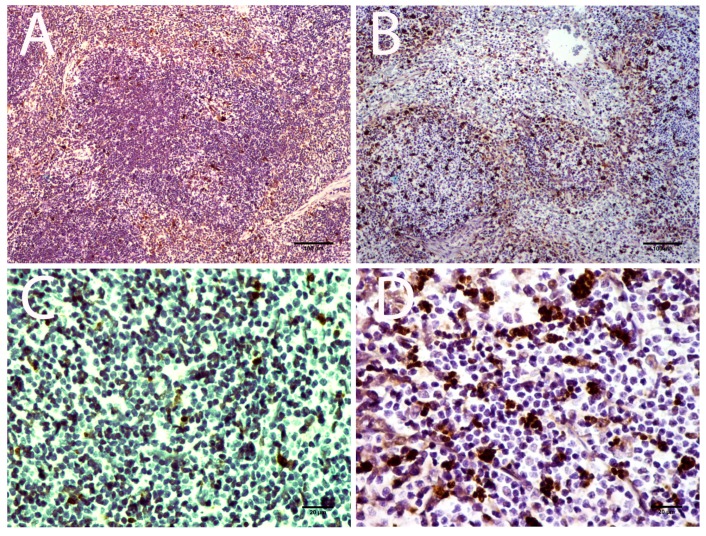
TUNEL assay in spleen sections. (**A**) Control animal: 100× magnification; (**B**) POWV-infected animal 100× magnification; (**C**) Control animal: 400× magnification; (**D**) POWV-infected animal: 400× magnification; (**A**,**B**) Scale bar corresponds to 100 μm; (**C**,**D**) Scale bar corresponds to 20 μm.

With the exception of the CNS, spleen and lymph nodes were the organs most infected with POWV in this study. Histopathological analysis of the spleen revealed white pulp hyperplasia, abundant tingible body macrophages in white pulp (Figure 6A,B), and many giant cells (Figure 6C,D). Tingible body macrophages were found throughout the spleen and were most notable in the white pulp. Giant cells were most common in the subcapsular areas of spleen tissue (Figure 6C,D). IHC for POWV antigen revealed virus in cortical and pericortical areas of the spleen, and tingible body macrophages were consistently immunostained (Figure 7). Since tangible bodies are associated with apoptosis, POWV induced cell death was suggested. The TUNEL assay confirmed that apoptosis in the spleen was prevalent, particularly within the tingible body macrophages (Figure 8).

**Figure 9:**
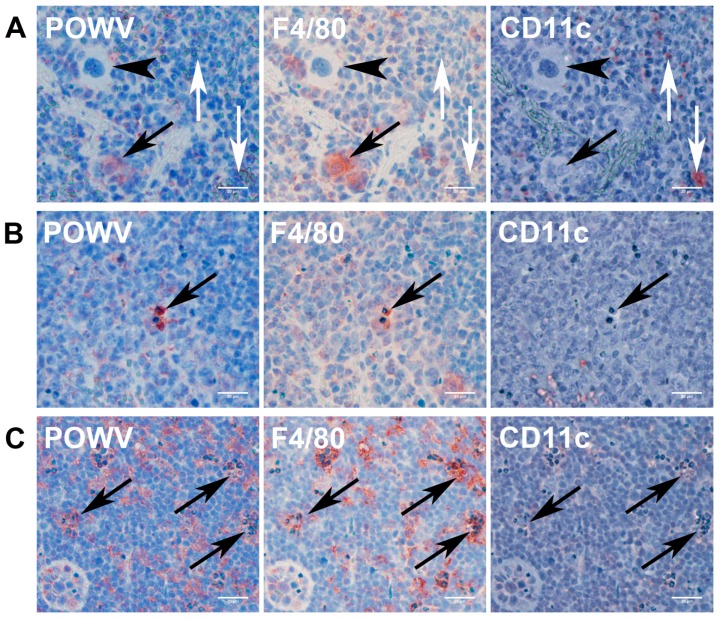
Multiple-step immunohistochemistry of POWV-infected spleen and lymph nodes sections. Successive immunohistochemistry reactions were performed using anti-POWV, anti-F4/80, and anti-CD11c antibodies. Panel A—POWV-infected animal, spleen focusing on the presence of giant cells and POWV positive macrophages; Panel B—POWV-infected animal, spleen focusing on tingible body bodies macrophages; Panel C—POWV-infected animal; lymph node. Black arrowheads indicate that giant cells are negative for all 3 stains. Black arrows indicate association of POWV and F4/80 staining but not CD11c. White arrows indicated CD11c staining; note that cells positive for CD11c are negative for both F4/80 and POWV staining. Scale bars correspond to 20 μm (400× magnification).

To identify POWV target cells in the spleen, we developed a sequential immunohistochemistry staining assay using AEC substrate. The sequential staining assay confirmed that splenic macrophages are the primary target cell for POWV infection (Figure 9, panels A–C). Furthermore, tingible body macrophages were positive for both F4/80 and POWV staining, further suggesting that they are infected macrophages (Figure 9, panel B). We cannot rule out the possibility that the POWV stained macrophages engulfed heavily infected cells. The giant cells that were present in the spleen did not stain for the virus, F4/80, or CD11c (Figure 9, panel A—arrowhead). The giant cells were also negatively stained for CD11b, a general myeloid cell marker (Figure S3). This suggested that these giant cells reflect a subset of F4/80- and CD11b-negative spleen macrophages. Viral antigen in lymph nodes also was present, and the positively stained cells were associated with the abundance of TUNEL-positive apoptotic cells in a pattern similar to the spleen infection. As in the spleen, macrophages were the main target of the virus (Figure 9, panel C). Bone marrow samples were present as part of the vertebra histology for spinal cord analysis. We did not detect any abnormalities in the bone marrow.

#### 3.3.3. Nonlymphoid Organs

No histopathological abnormalities were found in the kidney, pancreas, and muscle. The liver presented mild inflammation mainly observed as perivascular infiltration (Figure S4). No POWV immunostaining was detected in any of these organs.

## 4. Discussion

The development of animal models is a crucial step in the process of elucidating the pathophysiology of human diseases. Although animal models do not always reflect the pathogenesis of human disease, they are one of the most efficient ways to work towards effective treatments and vaccines. We examined the pathogenesis of Powassan virus (POWV) in a model that employed C57Bl/6 mice. This is the first study of a POWV animal model with a detailed neurovirological description of the CNS infection that also explores the potential pathogenic role of lymphoid organs. In this study, we have observed ventral horn infection of the spinal cord in mice, and this is consistent with the neuropathological changes and flaccid paralysis observed in humans.

Our data clearly demonstrate that C57BL/6 mice inoculated via footpad develop POWV infection with a clinical course similar to the human infection. Initial weight loss was quickly followed by behavior abnormalities, altered reflex, and paralysis. When humans are infected, paralysis is common and severe long-lasting sequelae is present in half of the survivors, including hemiplegia. Commonly, limb weakness or movement impairment precede other neurological signs and symptoms [9,10]. 

Post-mortem data of POWV infections in humans are scarce. The first available autopsy came from the index case. A 5-year-old boy developed encephalitis and suddenly stopped breathing and died. He presented with spastic hemiplagia and lost the right sided plantar reflex after four days of hospital care. Analysis of his brain showed meningoencephalitis with a dense infiltration of lymphocytes and macrophages [11]. Another post-mortem examination was done on a fatal case of DTV (POWV lineage II). That patient presented with fatigue, fever, rash, diplopia and weakness in the right leg and arm. Multiple lesions were observed using brain imaging including persistent hydrocephalus. The post-mortem examination of his CNS indicated infiltration of mononuclear cells in the spinal cord anterior horn and microglial nodules in the lateral corticospinal tracts and posterior columns. Mononuclear infiltrates with areas of necrosis and neuronophagia were identified in the brainstem, spinal cord, cerebellum, basal ganglia and thalamus, affecting mainly large motor neurons [8]. Gholan et al. [9] described muscle flaccidity and absence of reflex in one patient who died of cardiac arrest after 40 days of hospital admission for POWV encephalitis treatment. This patient also presented intense inflammation of meninges and brain parenchyma with microglial activation. Tissue inflammation and necrosis affected mainly basal ganglia and lower brainstem. Although this patient had signs of paralysis, no spinal cord samples were available in that case. Similarly, human autopsies from fatal TBEV (central European strain) cases indicated that severe illness is presented as a (myelo)meningoencephalitis with accentuated commitment of brainstem, spinal cord and cerebellum. Moreover, large neurons are preferably infected in the spinal cord anterior horns, medulla oblongata, pons, dentate nucleus, Purkinje cells, and striatum [40].

In the model that we have characterized herein, brainstem and spinal cord were the CNS sectors that were the most consistently infected. In the spinal cord, the ventral grey matter was the most affected with a strong POWV antigen detection by IHC. Vacuolization of the neuropil and microglia activation were common and the motor neurons of anterior horns were the main target for POWV infection. Paralysis of posterior limbs is a remarkable feature of POWV infection in both humans and animal models and is probably a consequence of viral infection of motor neurons in the ventral part of the spinal cord. Spinal cord necropsy indicated mononuclear infiltration accentuated in the anterior horn [8,11] and presence of virus was confirmed by sequencing [8]. Other neurological signs in our model included weak grip, altered reflex when grabbed by the tail, or walking/positioning abnormalities. These types of observations in the infected animals are analogous to the reports of areflexia and muscle weakness in human cases of POWV infection. Clinically and pathologically, damage of spinal cord is the most reasonable hypothesis to explain muscle weakness, areflexia and paralysis observed in humans and laboratory animals. Poliomyelitis-like paralysis is common for many encephalitic flavivirus infections, including West Nile Virus [41,42] and other encephalic tick-borne flaviviruses [43,44,45,46].

It is worth noting that both spastic and flaccid paralysis were described for POWV infections in humans. Mclean and Donohoe [11] described spastic hemiplegia with deep reflexes in the body right side in the POWV index case. Rossier et al. [47] also noticed spasticity in an 8 years-old child in 1972. Wilson et al. [48] described rigid right limbs in a 13 month-old infant and a case report from Tennessee (USA) described a 9-year old girl with rigidity in the upper extremities after POWV infection [49]. On the other hand, Gholan et al. [9] described flaccid paralysis in a case report of a 64 years-old man. Interestingly, all four cases above indicated as examples of spasticity after POWV infection were in young children but more detailed studies are necessary to verify if there is age-dependency for POWV induced spasticity. The presence of spasticity indicated that brain areas responsible for voluntary motor control might be affected in some cases [7,8,9,10,12,50]. 

Murine models are commonly used for neuropathogenesis studies. Frequently neurotropic viruses present paralysis as a hallmark of their infections and animal models are useful for the understanding of the mechanism that causes the paralysis. Poliovirus is the prototype of the viruses that cause flaccid paralysis. In transgenic mouse for the TgPVR21 receptor (poliovirus receptor), flaccid paralysis is caused by the infection of the brainstem nuclei and spinal cord, mainly motor neurons [51]. Flaccid paralysis studies have been investigated in mice for West Nile virus and reovirus serotype 3 infections [52,53,54]. In our study, we observed spinal cord ventral horn and brainstem infection with an important involvement of motor neurons. Motor neuron death in the spinal cord and brainstem is the probable cause of the flaccid paralysis observed. No spasticity was observed in animals used in our study.

Spleen and lymph nodes were the only organs outside of the CNS that exhibited significant histopathological changes. The lack of CNS infection in some individuals could therefore be linked with the lymphoid changes that may occur prior to infiltration of the virus into the CNS. Unfortunately, there is no autopsy data available that describes spleen infection in any POWV encephalitis cases. Previous analysis of the spleen in a BALB/c mouse model described white pulp activation, ill-defined germinal centers with tingible body macrophages, and white pulp populated by large blast-like cells; however, no POWV positive cells were detected [17] and this previous publication did not examine lymph nodes. The absence of POWV staining in the BALB/c model [14] can be a mouse lineage peculiarity or a difference in the sensitivity of the immunoassay. In our study, a high level of tingible body macrophages was present in both lymphoid organs. TUNEL assay together with sequential IHC staining suggested a virus-induced apoptosis mechanism in infected macrophages. Other histopathological alterations such as the presence of giant cells and white pulp hyperplasia were also noticed, but their importance in POWV pathogenesis requires further studies. Macrophage tingible body infection by TBEV complex viruses is not surprising. Macrophage lineages are susceptible to TBEV infection generating viral progeny in high titer [55,56] and primary cultured peritoneal macrophages gave similar infection rate for TBEV [57]. The importance of macrophages for TBEV complex members is corroborated by in vivo studies: mice inoculated peripherally demonstrated presence of TBEV antigen in macrophages at the tick bite site [58]. Moreover, TBEV can infect lymph nodes after 6 h post subcutaneous inoculation and this leads to the primary viremia [19,59].

Giant cells in spleen tissue were negative for POWV staining and are probably present as an indirect response to POWV infection. Interestingly, IHC for macrophage marker F4/80 and the general myeloid cell marker CD11b both failed to stain giant cells. Macrophages have the ability to fuse their membranes to large phagocyte antigens and are the main candidate to form giant cells [60,61]. Macrophage surface marker expression is influenced by the activation state (M1 or M2) [62] or by the tissue where this macrophage resides [63]. The spleen has four regional subtypes of macrophages [63,64]: (1) marginal zone macrophages; (2) metallophilic macrophages; (3) red pulp macrophages; and (4) white pulp macrophages. Only red pulp macrophages are known to express F4/80. Thus, we suggest that the giant cells present here are a type of macrophage that lacks F4/80 and CD11b staining. The phenotypic characterization of this macrophage subpopulation is an important issue for future studies using this model.

In summary, this study adds additional knowledge to our understanding of the pathogenesis of POWV infection in a mouse model. Our data indicate that: (1) neurons are the dominant infected cell type in the brain; (2) the brainstem is the most vulnerable sector of the CNS; (3) the ventral spinal cord is consistently infected in all animals and produces a poliomyelitis-like syndrome; (4) lymphoid organs are consistently infected tingible body suggesting an importance of these organs in POWV pathogenesis; (5) macrophages are the cells that appear to be infected in lymphoid tissue and their infection may result in apoptosis. This animal model system will be useful for future studies of POWV neurotropism and neuropathogenesis. 

## Supplementary Materials

The following are available online at www.mdpi.com/1999-4915/8/8/220/s1, Figure S1: Immunohistochemistry for CD11b detection in the Brain; Figure S2: Immunohistochemistry for GFAP detection in brain sections; Figure S3: Immunohistochemistry for CD11b detection in infected spleen section; Figure S4: Histopathological findings in the liver (HE staining), and Table S1: Summary of POWV pathological observations.
