# Supplementary material for: Spinal Cord Ventral Horns and Lymphoid Organ Involvement in Powassan Virus Infection in a Mouse Model
Source: Viruses. 2016 Aug 12;8(8):220. doi: 10.3390/v8080220 (PMC4997582; doi:10.3390/v8080220)
Supplement: Supplementary file 1 [file viruses-08-00220-s001.pdf]

Supplementary Materials: Spinal Cord Ventral Horns and Lymphoid Organ Involvement in Powassan Virus Infection in a Mouse Model

Rodrigo I. Santos, Meghan E. Hermance, Benjamin B. Gelman, Saravanan Thangamani

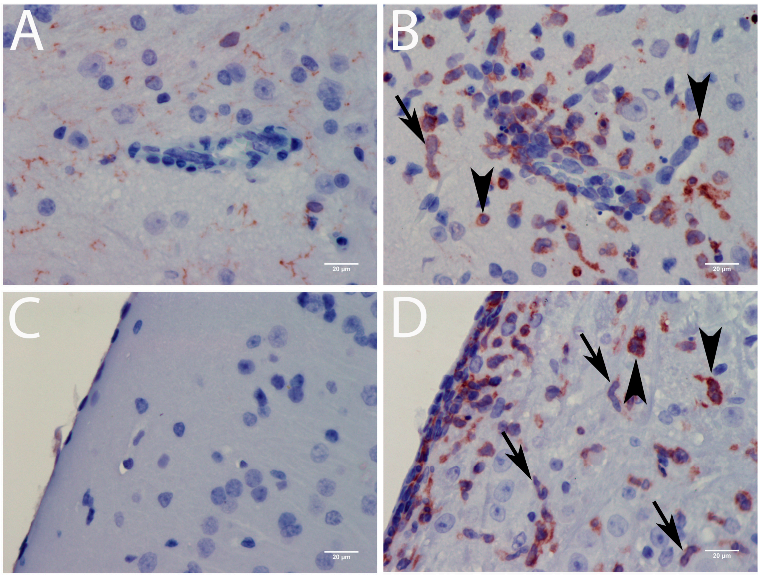

Figure S1. Immunohistochemistry for CD11b detection in the Brain. A) Control animal: vessel; B) POWV infected animal: perivascular infiltration; C) Control animal: meninges D) POWV-infected animal: meningitis. Scale bar correspond to 20µm (400X magnification). Arrows indicate microglial cells (rodcells), arrowheads indicate inflammatory macrophages.

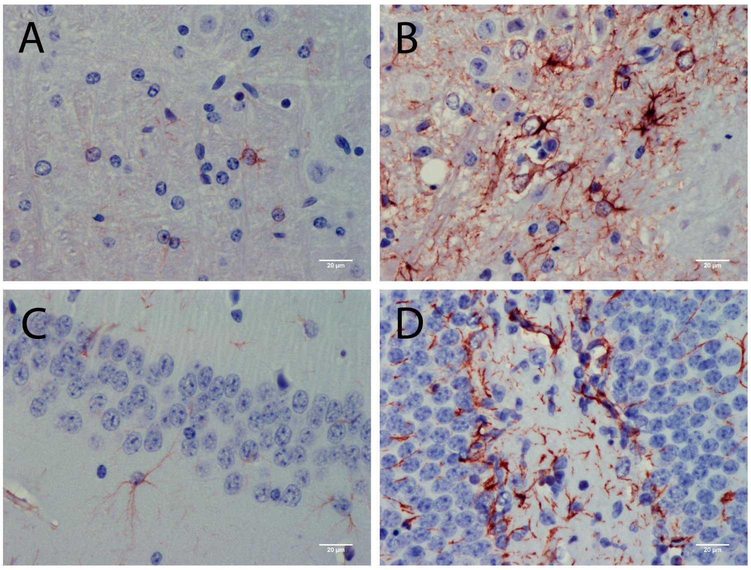

Figure S2. Immunohistochemistry for GFAP detection in brain sections. A)Control animal; normal GFAP staining for brainstem ; B) POWV infected animal: GFAP super-expression demonstrating astrogliosis in the brainstem; C) Control animal: normal GFAP staining for the hippocampus . D) POWV infected animal: GFAP super-expression demonstrating astrogliosis in the hippocampus. Scale bars correspond to 20µm (400X magnification).

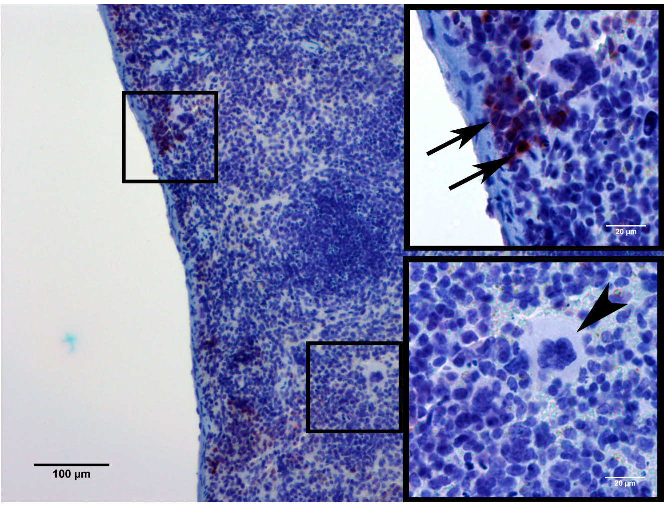

Figure S3. Immunohistochemistry for CD11b detection in infected spleen section. Arrows indicate CD11b-positive staining. Arrowhead indicates one giant cell; Note that giant cells are not stained for CD11b.

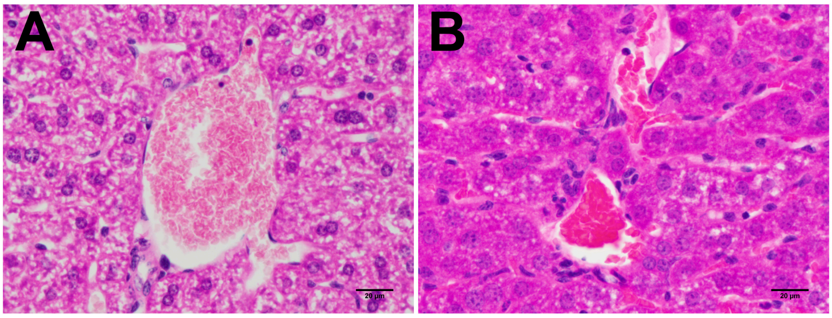

Figure S4. Histopathological findings in the liver (HE staining). A) Control animal; B) POWV-infected animal. Scale bars correspond to 20μm (400X magnification).

Table S1. Summary of POWV pathological observations

			Control				Low dose infection								High dose infection							
POWV staining	Cerebellum	Purkinge cells					Orange		Orange					Yellow	*		Orange	Yellow	Red	Orange	Red	Orange
		Granular					*													*		
		Molecular					Orange												Yellow	Yellow		
	Brainstem						Red		Red	Red	Orange	*	Yellow	Yellow	Black	Orange	Black	Orange		Black	Black	Yellow
	Hippocampus	CA1-4					Yellow	Red												Yellow		Orange
		Dorsal Ganglia					Yellow	Black												*		Yellow
	Thalamus						Black	Red	Red		Yellow	*	Orange	Yellow	Red	Yellow	Red			Black	Yellow	Black
	Midbrain							Red											Red	Red		Black
	Neocortex	Caudal					Red	Orange	Red		Yellow		Orange	*	Orange	Yellow				Red	Yellow	Yellow
		Medial					Red	Orange	Orange		Yellow				Orange			Orange	Yellow	Red	Yellow	Orange
		Frontal/piriform					Red		Black		Yellow				Red	Yellow		Yellow	Orange	Black	Yellow	Orange
	Basal Ganglia						Orange	Red	Red		Yellow				Orange	Yellow			Black	Black	Red	Red
	Corpus callosum						*															
Inflammation	Cerebellum	Purkinge cells					Orange											Yellow				
		Granular																				Yellow
		Molecular					Orange															Yellow
	Brainstem						Orange		Orange	Yellow	Yellow	Yellow	Yellow		Red		Red	Yellow	Red	Black	Black	Red
	Hippocampus	CA1-4					Yellow	Red	Yellow	Yellow			Yellow	Yellow					Yellow	Orange	Orange	Black
		Dorsal Ganglia					Orange	Red	Yellow	Yellow			Yellow							Orange	Orange	Black
	Thalamus								Red		Orange	Yellow		Yellow	Red		Red	Yellow		Red	Orange	Black
	Midbrain															Yellow						Black
	Neocortex	Caudal					Red	Red	Black	Yellow		Yellow	Orange	Yellow	Black	Orange						
		Medial					Red	Red	Black			Yellow		Yellow	Black	Yellow	Orange	Orange	Orange	Orange	Orange	Red
		Frontal/piriform					Red	Red	Black			Yellow		Yellow	Black		Red	Orange	Orange	Orange	Orange	Red
	Basal Ganglia						Yellow	Red	Black	Yellow	Yellow				Black	Orange						Red
	Corpus callosum																		Red			

Results are shown as POWV staining (upper panel) or inflammation (below panel) ranking between the groups: "white" no positive cells/no inflammation, * 1 to 5 positive cell in an area. The colors in each spot mean the grade of POWV-positive cells/inflammation for each brain area, where the yellow represents low level inflammation/POWV staining and the black represents wide spread inflammation/POWV staining. The color grade ranking from low to high are: Yellow, Orange, Red, Black

© 2016 by the authors, licensee MDPI, Basel, Swizerland. This article is an open access article distributed under the terms and conditions of the Creative Commons Attribution (CC-BY) license (http://creativecommons.org/licenses/by/4.0/).
